# Diffusion-Weighted MRI in Patients with Testicular Tumors—Intra- and Interobserver Variability

**DOI:** 10.3390/curroncol29020071

**Published:** 2022-02-02

**Authors:** Malene Roland Vils Pedersen, Martina Kastrup Loft, Claus Dam, Lone Ærenlund Lohmann Rasmussen, Signe Timm

**Affiliations:** 1 Department of Radiology, Vejle Hospital, University Hospital of Southern Denmark, 7100 Vejle, Denmark; martina.kastrup.loft@rsyd.dk (M.K.L.); claus.dam@rsyd.dk (C.D.); lone.aerenlund.lohmann.rasmussen@rsyd.dk (L.Æ.L.R.); 2 Department of Radiology, Kolding Hospital, University Hospital of Southern Denmark, 6000 Kolding, Denmark; 3 Department of Regional Health Research, University of Southern Denmark, 5230 Odense, Denmark; signe.timm@rsyd.dk; 4 Research Unit, Kolding Hospital, University Hospital of Southern Denmark, 6000 Kolding, Denmark

**Keywords:** DWI, ADC, region of interest, testicular tumor, interobserver

## Abstract

In general, magnetic resonance (MR) diffusion-weighted imaging (DWI) has shown potential in clinical settings. In testicles parenchyma, the DW imaging helps differentiate and characterize benign from malignant lesions. Placement and size of the region of interest (ROI) may affect the ADC value. Therefore, the aim of this study was to investigate the intra- and interobserver variability in testicular tumors when measuring ADC using various types of regions of interest (ROI). Two observers performed the ADC measurements in testicular lesions based on three ROI methods: (1) *whole volume*, (2) *round*, and (3) *small* sample groups. Intra- and interobserver variability was analyzed for all ROI methods using intraclass correlation coefficients (ICC) and bland-altman plots. The two observers performed the measurements twice, three months apart. A total of 26 malignant testicle tumors were included. Interobserver agreement was excellent in tumor length (ICC = 0.98) and tumor width (ICC = 0.98). In addition, intraobserver agreement was excellent in tumor length (ICC = 0.98) and tumor width (ICC = 0.99). The *whole volume* interobserver agreement in the first reading was excellent (ICC = 0.93). *Round* ADC had an excellent (ICC = 0.93) and fair (ICC = 0.58) interobserver agreement, in the first and second reading, respectively. Interobserver agreement in ADC *small* ROIs was good (ICC = 0.87), and good (ICC = 0.78), in the first and second reading, respectively. Intraobserver agreement varied from fair, good to excellent agreement. The ROI method showed varying inter- and intraobserver agreement in ADC measurement. Using multiple *small* ROI conceded the highest interobserver variability, and, thus, the *whole volume* or *round* seem to be the preferable methods.

## 1. Introduction

Diffusion-weighted imaging (DWI) is a magnetic resonance imaging (MRI) non-invasive technique measuring diffusion of molecular water. DWI may be used for evaluation of tumors and has shown potential in clinical settings. During the last decade, DWI, including apparent diffusion coefficient (ADC), has been applied in discriminating malignant from benign nodules in a variety of organs [1,2,3,4,5]. In general, adding DWI sequences to existing MR protocols is not a complicated and time comsuming task and can easily be applied to existing protocols.

Ultrasound is the first-choice modality when investigating testicles due to low cost, availability, and diagnostic accuracy. However, ultrasound is limited by operator dependency. MRI imaging is becoming popular as new features, such as ADC measurements, that have potential to impact in the diagnostic field. In addition, MRI has the ability to image small structures [6] and can be used as both a clinical and a problem-solving tool [7,8]. DWI imaging may add valuable information and is currently being applied when investigating various testicular conditions and diseases, e.g., microlithiasis, torsion, undescended testis and varicocele [1,9,10,11].

The Scrotal and Penile Imaging Working Group by European Society of Urogenital Radiology (ESUR) recommends additional scrotal MRI to characterize testicular masses [12], including T1, T2 and DWI sequences with at least three b-values. The Scrotal and Penile Imaging Working Group recommends use of scrotal MRI when ultrasound findings are inconclusive or inconsistent with clinical examination, when performing local staging of testicular malignancy, when differentiating benign from malignant tumors, and when assessing of acute scrotum [13].

In testicular parenchyma, DWI can differentiate and characterize benign from malignant tumors [1,14]. Tumours often display a heterogeneous appearance. The ADC measurement may depend on the selected region of interest (ROI). Therefore, choice of ROI shape may affect the ADC value. Few studies have investigated MRI interobserver variability in testicular tumors using different ROI size [14,15,16,17,18]. Tsili et al. investigated interobserver variability in testicular tumors using ADC and found DWI ADC interobserver variability to be excellent regarding ROI shape [14]. 

To our knowledge, no studies have evaluated the intra-observer variation of ROIs in testicular tumors. The aim of this study was, firstly, to assess the intra- and interobserver variability in ADC values using three different methods of ROI selection in testicular tumors and, secondly, to evaluate the measurement of MRI testicular length and width, including intra- and interobserver variability.

## 2. Materials and Methods

This retrospective study was approved by the local Danish Data Protection Agency (2012-58-0018) and the local hospital review board. Approval by the Regional Committee on Health Research Ethics was not required due to the retrospective study design. However, written informed consent was mandatory prior to MRI examination.

### 2.1. Patients

We retrospectively evaluated a total of 26 patients with malignant testicular tumors in the period 2013–2016. Only patients with histopathological confirmed malignant testicular tumor were included. The National Pathology Registry database was searched [19] using the Danish unique civil registration number [20]. We excluded one patient, as the MRI investigation was performed after the orchiectomy.

### 2.2. MRI

The MRI examination was performed using an Ingenia 1.5-Tesla system (Philips Medical Systems, Best, Netherlands) with patients in prone position using the posterior coil placed at the center of the scrotum. A T2-weighted spin echo, T1-weighted and DWI sequences with b values of 0, 100, 300, 600, 900 and 1100 s/mm^2^ were performed, and ADC maps were generated automatically at the workstation. The MRI protocol has been published previously and details are presented in Table 1 [1]. All data was stored in the hospital’s Picture Archiving and Communication System (PACS). 

### 2.3. Observers

The two observers individually interpreted the 26 malignant testicular tumors. The two observers measured tumor length and width on the T2-weighted images. Two senior radiologists with 8–10 years of experience participated in this study. The two observers used the same type of diagnostic screen (21.3 monitor CCL358i2 from TOTUKU, JVCENWOOD Cooperation, Kanagawa, Japan) to review all 26 cases. All images were viewed using an Easyviz Impax PACS workstation (Medical Insight, Valby, Denmark).

The observers were placed in an undisturbed office and were unable to discuss the cases with colleagues. The observers independently measured the ADC on 26 testicular tumors using the three ROI types and were blinded to histopathological findings, previous examinations and patient medical history. The observers were blinded to each other’s measurements.

**Figure 1:**
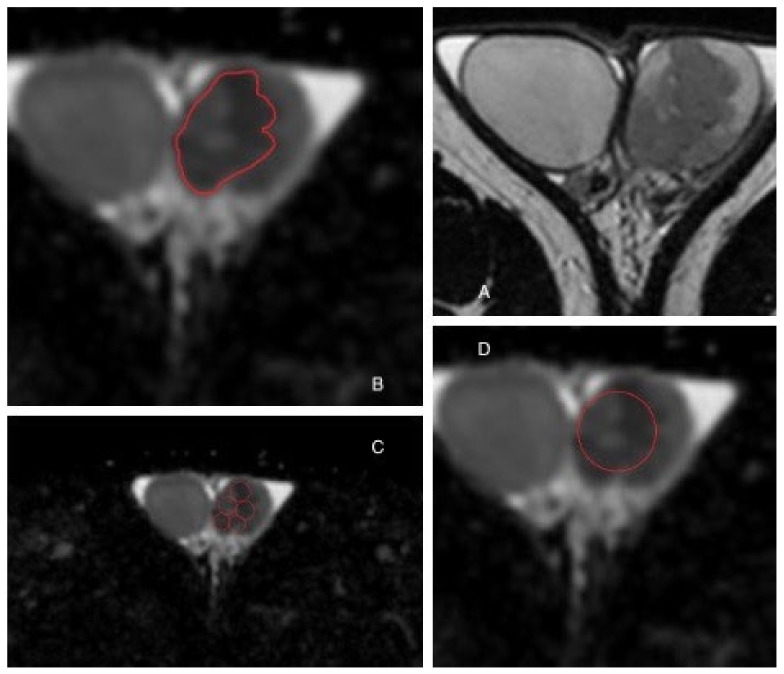
Testicular tumor in a 36-year old male. (**A**) is an MRI of both testicles. (**B**) is *whole volume* ROI. (**C**) is *small* ROIs. (**D**) is *round* ROI.

The ROI was placed in one of the following positions: *whole volume*, *round* ROI and *small* ROI. The *whole volume* ROI was as large as possible, covering all the tumor tissue. The *round* ROI was placed in the center of the tumor as large as possible. The *small* ROIs were 3 mm in size and were placed in up to five individual places within the tumor without any overlap; a mean was calculated (Figure 1). In general, all ROIs were placed carefully to avoid overlap with other testicular tissue. 

To limit observer bias, the two observers re-evaluated the 26 testicular tumors after a period of 3 month. Cases were presented in random order. 

### 2.4. Statistical Analysis

Intra- and interobserver absolute agreement of length, width, and tumor ADC value in the three ROI methods was assessed by interclass correlation coefficients (ICC). A two-way random effect absolute agreement model was used to estimate the interobserver ICC, including 95% confidence intervals (95% CI). A two-way mixed effect absolute agreement model was used for calculating the ICC intraobserver agreement. The ICC were interpreted as poor (below 0.50), fair (0.50–0.75), good (0.76–0.90) and excellent (above 0.90) [21]. 

Bland–Altman plots were used to assess the intraobserver agreement across the scale on testicular tumor length, width, and ADC measurements by plotting the differences between the two readings against the mean of each test-retest measurement pair. 

Since data were not normally distributed, limits of agreement (LOA) were estimated as non-parametric using 2.5th and 97.5th percentiles. Statistical analyses were carried out with STATA statistical software (version 17.0 STATA, College Station, TX, USA).

## 3. Results

Figure 1 shows placement of the three ROI methods within a testicular tumor.

The patients median age was 38 years (range 23–79 years). All tumors were confirmed by histopathology. Main MRI scan parameters are presented in Table 1. Descriptive statistics on ADC, tumor length and width assessed by the two observers using the three ROI methods are presented in Table 2 and Table 3.

### 3.1. Interobserver Agreement (between Two Observers)

#### 3.1.1. Tumor Length and Width

The interobserver agreement in tumor length was excellent in the first reading ICC = 0.98 (95% CI 0.93–0.99) and second reading ICC = 0.98 (95% CI 0.94–0.99). Tumor width interobserver agreement was excellent in the first and second reading with ICC = 0.98 (95% CI 0.92–0.99) and ICC = 0.98 (95% CI 0.95–0.99), respectively.

#### 3.1.2. Whole, Round, Small ROI

In the first reading, the ADC *whole volume* ROI showed excellent agreement (ICC = 0.93, 95% CI 0.86–0.96) and good agreement in the second reading (ICC = 0.89, 95% CI 0.78–0.95).

*Round* ADC showed excellent agreement with an ICC = 0.93 (95% CI 0.85–0.97) and fair agreement in the second reading (ICC = 0.58, 95% CI 0.25–0.78). The interobserver agreement in ADC *small* ROIs was good in the first and second reading with ICC = 0.87 (95% CI 0.72–0.94) and ICC = 0.78 (95% CI 0.54–0.90), respectively. Tumor size was heterogene (Table 3).

### 3.2. Intraobserver Agreement (within Observers)

The intraobserver agreement for observer 1 was excellent for tumor length (ICC = 0.98, 95% CI 0.93–0.99) and tumor width (ICC = 0.99, 95% CI 0.98–0.99).

Intraobserver variability (observer 1) showed good to excellent agreement for *whole volume* ROI (ICC = 0.95, CI 0.89–0.97), fair agreement in *round* ROI (ICC = 0.71, CI 0.45–0.86), and fair agreement in *small* ROIs (ICC = 0.71, CI 0.41–0.86).

Observer No. 2 showed excellent intraobserver agreement in tumor length (ICC = 0.99, CI 0.98–0.99) and width (ICC = 0.99, CI 0.97–0.99). In addition, observer 2 showed good to excellent agreement in the three ROI methods *whole volume* (ICC = 0.86, CI 0.72–0.93)*, round* (ICC = 0.93, CI 0.84–0.97) and *small* ROIs (ICC = 0.97, CI 0.94–0.97).

**Figure 2:**
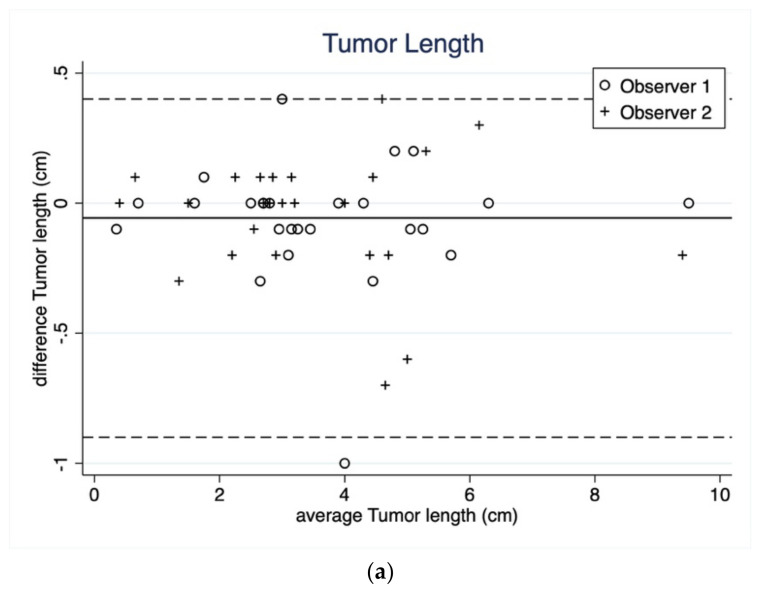

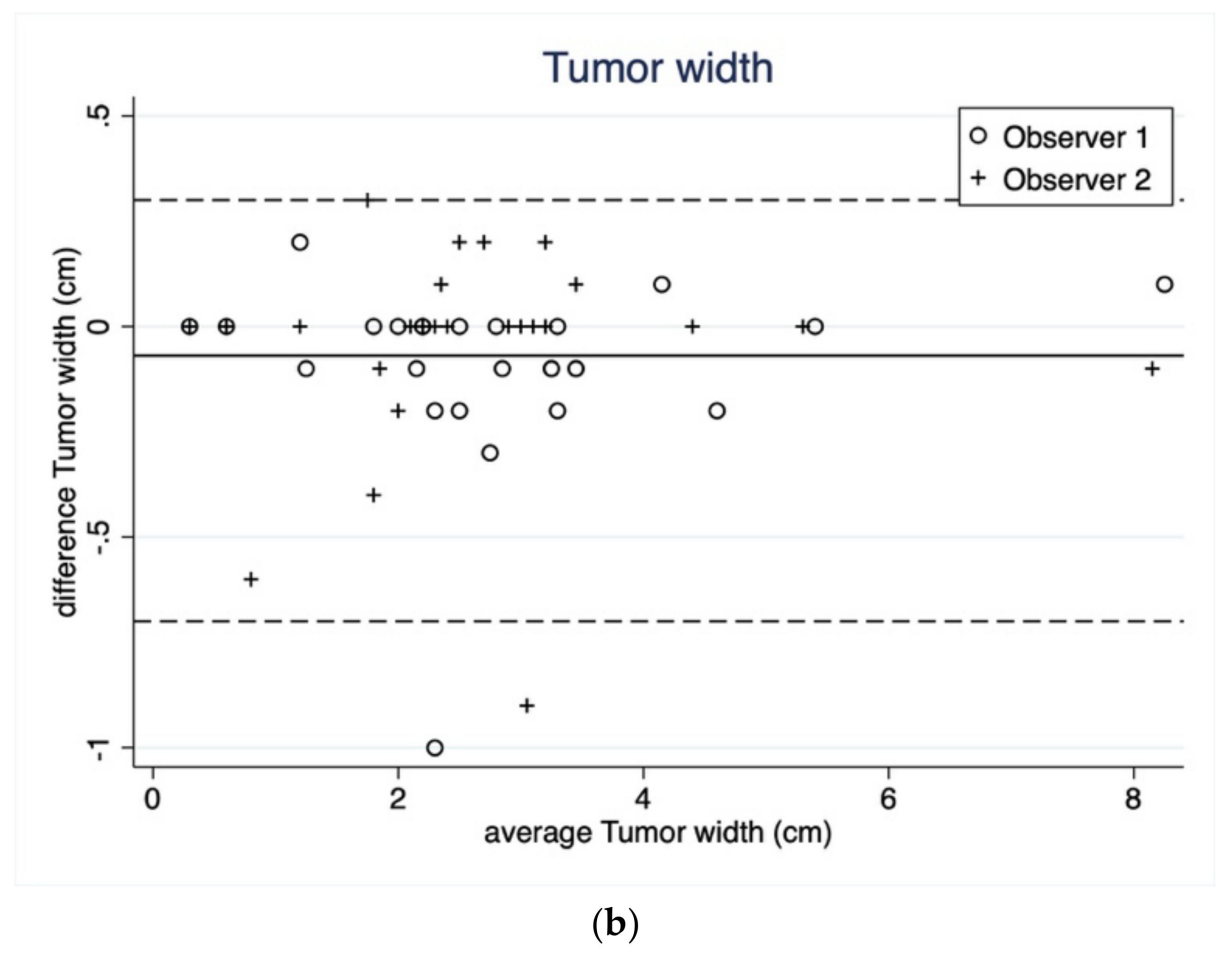
(**a**) Bland–Altman plot showing intraobserver agreement as average tumor length in reading 1 and 2 plotted against mean tumour length of the two readings separated on observer ID. The bold line corresponds to the mean difference (−0.06 cm) and the dashed lines corresponds to the upper and lower 95% limits of agreements (LOA) (95% LOA 0.40 to −0.90 cm). (**b**) Bland–Altman plot, showing intraobserver agreement as average tumor width in reading 1 and 2 plotted against mean tumour width of the two readings separated on observer ID. The bold line corresponds to the mean difference (−0.07 cm) and the dashed lines correspond to the upper and lower 95% limits of agreements (95% LOA 0.27 to −0.71 cm).

**Figure 3:**
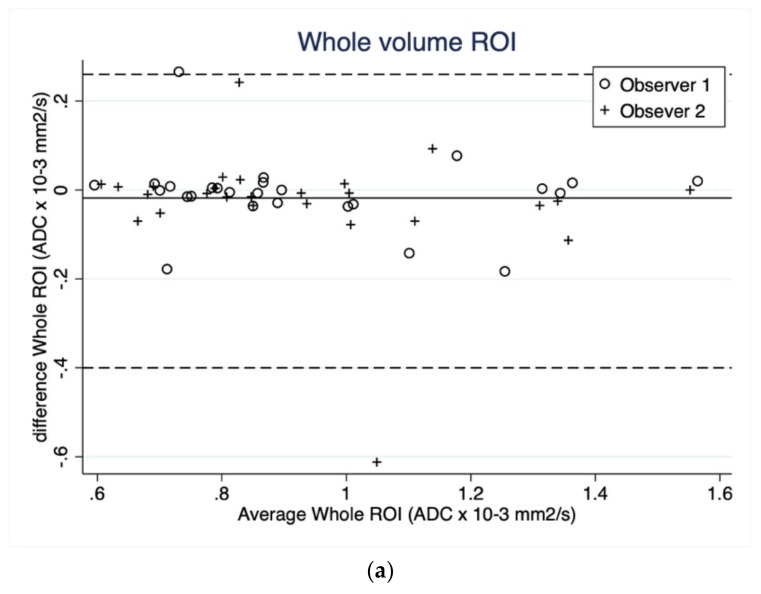

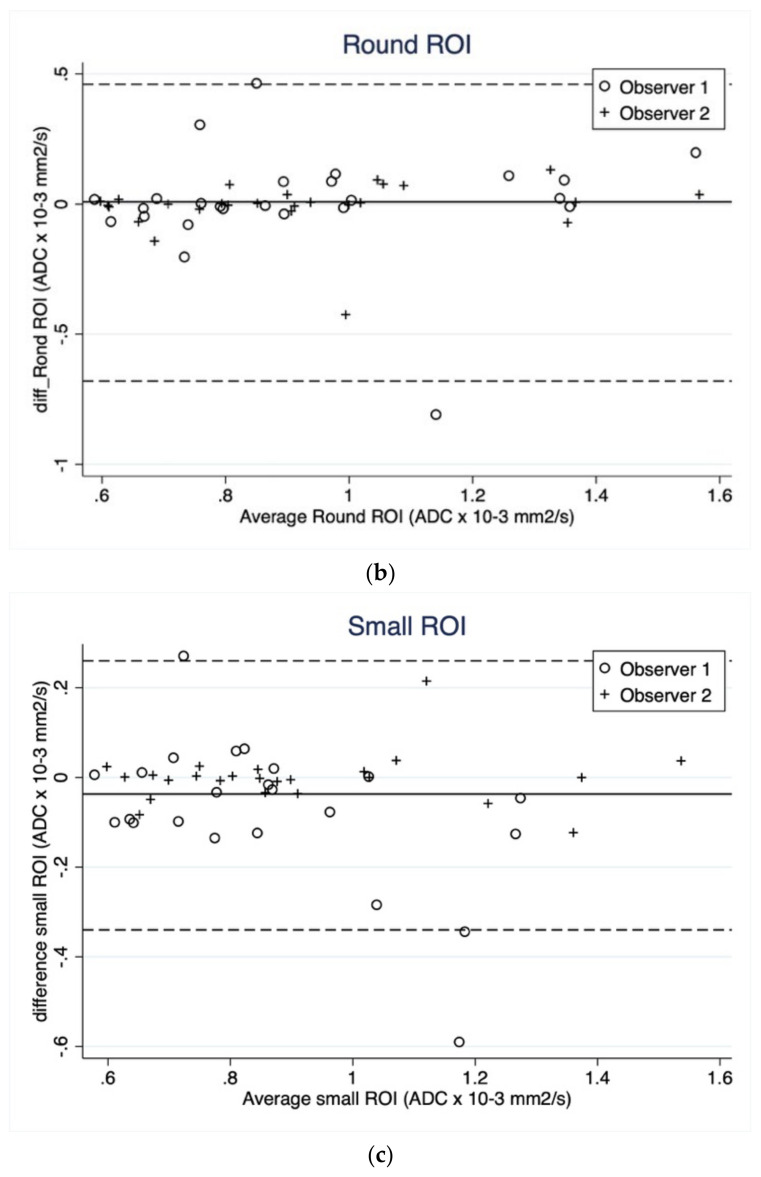
(**a**) Bland–Altman plot showing intraobserver agreement as average tumor apparent diffusion coefficient (ADC) measured by whole tumor volume in reading 1 and 2 plotted against mean tumour ADC measured by *whole volume* of the two readings separated on observer ID. The bold line corresponds to the mean difference (−0.02 × 10^−3^ mm^2^/s) and the dashed lines correspond to the upper and lower 95% limits of agreements (95% LOA 0.26 to −0.40). (**b**) Bland–Altman plot showing intraobserver agreement as average tumor apparent diffusion coefficient (ADC) measured as regions of interest (ROI) *round* shape in reading 1 and 2 plotted against mean tumour ADC measured as ROI *round* shape of the two readings separated on observer ID. The bold line corresponds to the mean difference (−0.01 × 10^−3^ mm^2^/s) and the dashed lines corresponds to the upper and lower 95% limits of agreements (95% LOA 0.46 to −0.68). (**c**) Bland–Altman plots showing intraobserver agreement as average tumor apparent diffusion coefficient (ADC) measured as region of interest (ROI) small in reading 1 and 2 plotted against mean tumour ADC measured as ROI small of the two readings separated on observer ID. The bold line corresponds to the mean difference (−0.04 × 10^−3^ mm^2^/s) and the dashed lines corresponds to the upper and lower 95% limits of agreements (95% LOA 0.21 to −0.29).

Bland–Altman plots on intraobserver agreement for tumor length, width and the three ROI ADC measurements are shown in Figure 2a,b and Figure 3a–c. Bland–Altman plots on length and width showed a mean difference of −0.06 (95% LOA 0.40 to −0.90) and −0.07 (95% LOA 0.27 to −0.77), respectively.

Bland–Altman plots on the three ROI methods of ADC measurement showed a better absolute agreement in ROI *whole volume* (mean difference = −0.02, 95% LOA 0.26 to −0.40) and ROI *round* (mean difference = −0.01, 95% LOA 0.46 to −0.68) than ROI *small* (mean difference = −0.04, 95% LOA 0.26 to −0.34). In general, the Bland–Altman plots suggested no systematic bias in terms of varying extend of agreement along the measurement scale.

The mean difference in all the ROI methods was very close to 0 (ranging from −0.01 × 10^−3^ mm^2^/s in *round* ROI ADC to 0.07 cm tumor width) indicating excellent absolute agreement. However, *round* ROI ADC showed the highest LOA (95% LOA 0.46 to −0.68 × 10^−3^ mm^2^/s) compared to *whole volume* ROI and *small* ROI.

## 4. Discussion

Our results show that the reliability of intraobserver measurements in testicular tumor length and width was excellent in both readings. The *whole, round* and *small* ROI method ranged from good to excellent agreement in the two reading.

MRI of the scrotum provides valuable information and DWI can improve the diagnostic accuracy in testicular tissue [1,14,22,23,24,25]. However, it is of great importance that the ADC measurements have a reproduceble interobserver variability [14]. Other studies with focus on ROI shape, ROI placement, and ADC measurements in various organs have good observer reproducibility [26,27,28,29,30].

The excellent level of interobserver agreement shown in this study may be explained by the similar level of experience by the two observers. However, measurement repeatability is important, regardless of experience. Still, variability may be related to shape and size of the ROI. Clauser et al. pointed out that using a small ROI placed only in the lowest signal intensity area versus an ROI containing the whole lesion may provide variability [28]. Furthermore, Inoue et al. found significant values in endometrial cancer using various ROI methods with the same size [31]. In rectal cancer Lambregts et al. found significant variability when using different ROI sizes, with whole-volume ROI showing the best reliability [29]. Zhou et al. investigated benign and malignant thyroid nodules and found ROI methods to affect the ADC values, with whole-volume ROI showing the best reliability [32].

In testicular lesions, Tsili et al. found excellent interobserver agreement regardless of ROI shape and size [14], which was also the case in our study, indicating that the organ placement, appearance, and accessibility may positively affect the variability.

In this study, the observers used three ROIs methods: *whole volume*, *round* and *small* ROIs. In general, it is important to know that the choice of ROI type can affect the outcome. Priola et al. investigated ADC whole-volume and found whole tumor volume to provide the most reproducible results in patients with advanced rectal cancer [33]. We found *whole volume* was the most reproducible method.

We used a total of six b-values (0, 100, 300, 600, 900 and 1100 s/mm^2^), which is considered a high number. B-values measure the degree of diffusion weighting applied to the image. The ADC is calculated by the applied b-values, usually two or more. By adding more b-values to the DWI, the scan time is increased. Kuhnke et al. advocates that ADC values can be calculated using two b-values [34]. Many papers choose to include two b-values, as the gain by adding more b-values is relatively small. Still, it is important to consider the number and size of b-values, as the ADC values will be affected by this choice.

### Strengths and Limitations

There are some limitations in the present study. MRI scans were all performed on the same MRI 1.5-tesla unit, which is considered a strength. It is possible that a 3-tesla unit could have achieved an improved image quality and, hence, the observers may have had better visualization of the testicular tissue and potentially better placement of the ROIs within the malignant lesion. However, this seems unlikely, as both 1.5 and 3 tesla units provides prominent images. The number of patients included (*n* = 26) and number of radiologist (*n* = 2) is limited; however, other studies have included the same number of radiologists and patients. We used three types of ROIs: *Whole volume*, *round*, and *small*, assessed by two senior observers; we consider it a strength to include different types of ROIs, as radiologists typically have dissimilar ROI preferences. Furthermore, it is a strength that all patients included had histopathological confirmed testicular cancer. However, currently, there is no generally accepted ADC differnce to compare the LOA with, which makes it difficult to compare results. In this study, we found low LOA, e.g., ADC −0.68.

## 5. Conclusions

This study demonstrates that all three ROI methods can be used when measuring ADC values in malignant testicular tumors; however, we found the *whole volume* ROI to be the most reproducible method. The intra- and interobserver agreement in tumor length and width was excellent, and ROI *whole volume*, *round*, and *small* ranged from good to excellent agreement.

## Figures and Tables

**Table 1:** Magnetic resonance imaging parameters and sequences for scrotum evaluation [1].

MRI Sequence	TR (ms)	TE (ms)	FOV (mm^2^)	Matrix	Spacing (mm)	Slice (mm)
Axial T1	650	10	110 × 110	140 × 137	0.9	2
Axial T2	2593	100	130 × 130	164 × 162	0.9	2
Axial DWI	3224	108	200 × 200	124 × 122	0.4	3

Abbreviations: DWI = diffuison weighted imaging, TR= Time to repeat, TE = time to echo, FOV= field of view.

**Table 2:** Data from the two readings shows the apparent diffusion coefficient (×10^−3^ mm^2^/s) measurements by *whole volume*, *round* and *small* region of interests independently by two observers (*n* = 26).

	Parameters	Whole Volume ROI		Round ROI		Small ROIs	
		Observer 1	Observer 2	Observer 1	Observer 2	Observer 1	Observer 2
Reading 1	ADC	0.860	0.862	0.859	0.899	0.842	0.862
	Q1–Q3	0.764–1.111	0.740–1.081	0.708–1.135	0.759–1.016	0.688–1.013	0.728–1.016
	Range	0.590–1.554	0.600–1.552	0.579–1.545	0.592–1.549	0.575–1.469	0.586–1.518
Reading 2	ADC	0.870	0.841	0.894	0.873	0.855	0.851
	Q1–Q3	0.754–1.021	0.750–1.003	0.709–1.071	0.717–1.074	0.686–0.917	0.733–1.042
	Range	0.601–1.574	0.613–1.552	0.581–1.660	0.603–1.585	0.561–1.411	0.611–1.555

Abbreviations: ROI = Region of interest ADC = Apparent diffusion coefficient. ADC is displayed as median. Interquartile range; Q1 = 25th percentile, Q3 = 75th percentile.

**Table 3:** Median tumor length and width (*n* = 26) measured on T2 weighted. Measurements are presented in cm.

	Observer 1		Observer 2	
Length (cm)	Reading 1	Reading 2	Reading 1	Reading 2
Median	3.3	3.2	3.1	3.1
Q1–Q3	2.7–4.7	2.7–.4.8	2.6–4.5	2.6–4.5
Range	0.4–9.5	0.3–9.5	0.4–9.5	0.4–9.5
Width (cm)	Reading 1	Reading 2	Reading 1	Reading 2
Median	2.8	2.6	2.4	2.5
Q1–Q3	2.2–3.4	2.0–3.3	2.0–3.1	1.9–3.1
Range	0.3–8.2	0.3–8.3	0.3–8.2	0.3–8.2

Abbreviations: ADC = Apparent diffusion coefficient, interquartile range; Q1 = 25th percentile, Q3 = 75th percentile.

## Data Availability

Data sharing is not applicable to this article.
